# Dr. Geo. Coatsworth

**Published:** 1855-11

**Authors:** 


*Dr. Geo. Coatsworth.*—The first article in the Original department of the October number of this *Journal* appeared without the name of the author. It should have been credited to Dr. Geo. Coatsworth, whose brief residence in this city was sufficient to make for him many friends both in and out of the Profession.

